# Activity of combinations of bactericidal and bacteriostatic compounds in *Mycobacterium abscessus*-infected mice: an overview

**DOI:** 10.3389/fmicb.2025.1616149

**Published:** 2025-08-01

**Authors:** Alessio Lanni, Elisabetta Iona, Lanfranco Fattorini, Federico Giannoni, Angelo Iacobino

**Affiliations:** Department of Infectious Diseases, Istituto Superiore di Sanità, Rome, Italy

**Keywords:** *Mycobacterium abscessus*, drug combinations, mice, bactericidal drugs, bacteriostatic drugs

## Abstract

Treatment of *Mycobacterium abscessus* (MAB) infections is complicated by the lack of bactericidal antibiotics, the ability of MAB to persist in the hypoxic environment of granulomas and the intrinsic antibiotic resistance, which results in patient treatment with drug combinations for several months. Therefore, the search for new drugs/drug combinations is an urgent need. This review provides a comprehensive update on the activity in the lungs of MAB-infected mice of new and old bactericidal and bacteriostatic compounds, alone and in combination, which showed killing greater than or equal to clinically used antibiotics and combination components. The acute model (4–14 days of treatment) was preferred in most single-drug efficacy testing procedures while the chronic model (28–42 days) was primarily used for combinations. Overall, 15 of 17 new compounds and 13 of 14 combinations decreased MAB colony forming units (CFUs) more than comparator drugs or combination components. The most potent combinations were those formed by bactericidal agents (≥1−log_10_ CFU reduction compared to the initial bacterial burden), consisting of two β-lactams and a β-lactam plus a β-lactamase inhibitor. Among the other combinations, activity of the bactericidal compounds was usually slightly increased by the bacteriostatic agents that, however, preserved the bactericidal core of combinations and suppressed emergence of drug resistance. Overall, these data suggest that there is an urgent need for systematic *in vivo* investigations on anti-MAB activity of combinations containing bactericidal drugs that are part of current treatment guidelines or of new, preferably oral compounds, to ultimately eradicate non-replicating persisters at the sites of disease.

## 1 Introduction

Non-tuberculous mycobacteria (NTM) include approximately 200 species classified in two categories according to their growth rate, namely the slowly growing mycobacteria (SGM) (e.g., *Mycobacterium avium* complex (MAC), *Mycobacterium xenopi, Mycobacterium kansasii, Mycobacterium marinum*, and *Mycobacterium simiae*), and the rapidly growing mycobacteria (RGM) (e.g., *Mycobacterium abscessus* (MAB), *Mycobacterium fortuitum*, and *Mycobacterium chelonae*), requiring >7 days and ≤7 days, respectively, to produce colonies on solid media (Johansen et al., 2020; Ratnatunga et al., 2020; Giannoni et al., 2023). NTM are environmental microorganisms, with most of them being saprophytes or non-pathogenic to humans. The pathogenic species generate a spectrum of clinical manifestations including, for instance, lung infections (MAC, MAB*, M. xenopi*, and *M. kansasii*) and soft tissues infections as result of surgery interventions and/or accidental lesions (*M. fortuitum*, *M. chelonae*, and *M. marinum*). In the last years, increase in research into these once poorly known diseases and in molecular diagnostic tools raised the detection of NTM infections.

Given the abundance of NTM in the environment (soil, dust, and water-based aerosols), these organisms cause infections often undiagnosed and difficult to treat (Johansen et al., 2020; Ratnatunga et al., 2020). Patients at high risk of infections include people with (i) immune suppression due to primary immunodeficiency syndromes associated with IL12-p40, IL12, and IFNγ receptor abnormalities and gene deformities, (ii) genetic or acquired structural lung diseases like cystic fibrosis (CF), chronic obstructive pulmonary disease (COPD), non-CF bronchiectasis, lung cancer, (iii) acquired immunodeficiency syndromes including AIDS and hematological malignancies (Johansen et al., 2020; Ratnatunga et al., 2020).

In immunodeficient patients, about half of NTM diseases were pulmonary, and the remainder was split between skin/soft tissue and disseminated lesions. In autopsy-based series of NTM diseases, pulmonary NTM infections were associated with extensive bronchiectasis and cavity formation with bronchocentric granulomatous inflammation (O’Connell et al., 2012). In contrast, disseminated diseases related to defects of the IL-12/IFN-γ axis were accompanied by a paucity of lung findings. When the lung was involved, it was with poorly formed granulomas (O’Connell et al., 2012).

In immunocompetent individuals with COPD and CF, pulmonary pathology included nodular and fibrocavitary lesions reminiscent of TB lesions (Wu et al., 2018). Video-assisted thoracoscopic surgeries of NTM infections revealed multiple caseating granulomas with necrosis. Chest computed tomography (CT) was useful for radiologists and physicians for distinguishing pulmonary TB and MAC lung diseases when typical findings were observed (Miura et al., 2020). CT and chest radiographs showed that, in general, bronchiectasis was more frequently seen in NTM than in TB diseases, while cavities were found both in lungs of TB and NTM-infected patients (Miura et al., 2020; Yuan et al., 2014; Kim et al., 2014; Kim C. et al., 2017; Oshitani et al., 2020). Large cavity size on CT showed strong relationships with MAC disease progression, which led to respiratory failure and high mortality (Oshitani et al., 2020). Chronically MAC-infected mice also developed necrotizing granulomas with minimal microvessels, and hypoxic centers similar to those of TB (Wu et al., 2018; Aly et al., 2007).

A poor response to treatment was seen in NTM infections with fibrocavitary lung disease (Swenson et al., 2018). Pharmacotherapies of NTM diseases are detailed in various publications (Daley et al., 2020; Johnson et al., 2023), and are based on the drug susceptibility testing (DST) criteria of the Clinical Laboratory Standards Institute (Clinical and Laboratory Standards Institute [CLSI], 2018; Brown-Elliott and Woods, 2019). In general, except when resistance is documented by DST, oral treatment with azithromycin (AZT) or clarithromycin (CLR) is the backbone of most NTM therapies.

## 2 *Mycobacterium abscessus* infections

Among RGM, the MAB complex includes the subspecies *Mycobacterium abscessus*, *Mycobacterium massiliense*, and *Mycobacterium bolletii* (Johansen et al., 2020; Lanni et al., 2022). Human lungs with MAB infections develop well organized granulomas including central caseous necrosis with few or no cells containing inner caseum and a neutrophil rim at the outer caseum edge (Kaya et al., 2022). Central caseous necrosis is surrounded by lipid-loaded macrophages with an outer lymphocyte-reach cuff and collagen rim. These features are similar to those of human and rabbit TB (Kaya et al., 2022). MAB-induced granuloma formation is dependent on TNF-α signaling and neutrophil trafficking (Bernut et al., 2017). Similarly to *Mycobacterium tuberculosis* (MTB) infections, MAB persist intracellularly and extracellularly within patients, which remain asymptomatic for years, with mycobacteria being recovered in biopsy specimens collected from granulomas. *M. abscessus* may show a transition from the smooth to the rough colony morphotype during persistent infection, resulting in granuloma breakdown and formation of extracellular bacterial cords (Johansen et al., 2020). *M. abscessus* also forms biofilms within the thickened alveolar walls and lung cavities during pulmonary infections (Fennelly et al., 2016; Dartois and Dick, 2024a).

In MAB-infected patients, mycobacteria encounter conditions different from those met in aerated nutrient-rich broth used for the minimum inhibitory concentration (MIC) assays. Indeed, as in human TB, the hypoxic environment of granulomas may induce formation of non-replicating (NR) drug-tolerant dormant cells without genetic mutations (persisters) (Yam et al., 2020; Lanni et al., 2023). In this view, the MIC assay is a poor predictor of clinical outcome, because patients harbor bacilli ranging from actively replicating (AR) to NR states. Several investigators and our group developed *in vitro* MAB culture models of hypoxic conditions, nutrient starvation and biofilm for DST and/or metabolic/transcriptomic studies (Lanni et al., 2022; Yam et al., 2020; Miranda-CasoLuengo et al., 2016; Berube et al., 2018; Simcox et al., 2023). Non-replicating cultures were also reported for MAC (Lewis and Falkinham, 2015; Totani et al., 2017; Abukhalid et al., 2023) and *Mycobacterium smegmatis* (Dick et al., 1998; Berney and Cook, 2010; Drapal et al., 2016; Wu et al., 2016). The dormancy regulon DosR, that is crucial for MTB survival in hypoxia, was conserved across NTM species and was upregulated upon nitric oxide (NO)-induced hypoxia (Wu et al., 2018; Miranda-CasoLuengo et al., 2016). A study showed the importance of the MAB DosRS two-component system for adaptation to hypoxia, highlighting a distinct regulon including genes conserved across mycobacteria and MAB-specific genes (Simcox et al., 2023).

There is not therapy that efficiently cures MAB lung diseases which, due to the intrinsic drug resistance of MAB, shows cure rates of only 25%–58% (Dartois et al., 2024; Ganapathy and Dick, 2022). Current therapy of macrolide-susceptible cases is based on administration of oral CLR, AZT, clofazimine (CLF) or linezolid (LNZ), and parenteral drugs including amikacin (AMK) with either tigecycline (TGC), imipenem (IMP), or cefoxitin (CFX) (Daley et al., 2020; Ganapathy and Dick, 2022). Treatments last several months and may be associated with drug side effects, poor compliance and acquired drug resistance like, for instance, that generated by the *erm(41)*-inducible macrolide resistance. New chemical substances with either novel mechanisms of action or drug targets could address development of intrinsic and acquired drug resistance, treatment shortening, reduced side effects. A strategy to accelerate drug discovery is to screen advanced lead compounds against TB that indeed yield high hit rate when compared to a random compound library (Ganapathy and Dick, 2022). This approach may allow to identify promising molecules that could more efficiently populate the almost empty MAB clinical development pipeline (Dartois and Dick, 2024a; Ganapathy and Dick, 2022; Sriram et al., 2022; Egorova et al., 2021; Quang and Jang, 2021).

Here, we examined the literature to upgrade the stakeholders on new and repurposed compounds with activity in MAB-infected mice greater than positive control comparator drugs (Sriram et al., 2022) mostly represented by those recommended in clinical guidelines (Daley et al., 2020; Griffith and Daley, 2022; Haworth et al., 2017). While the mouse models have been important to predict drug efficacy against MTB and MAC, preclinical acute models (1–2 weeks treatment initiated 1–2 days postinfection) and chronic models (4 weeks treatment initiated 4 weeks postinfection) for testing drugs are more challenging because MAB murine infections are difficult to establish. Indeed, MAB is low virulent for immunocompetent mice, making necessary the use of immunodeficient mice models or specific routes of infections for drug testing in immunocompetent mice. In spite of these limitations, preclinical mice models are widely reported for investigating host-pathogen interactions and testing new drugs and their combinations (Ganapathy and Dick, 2022; Sriram et al., 2022; Rampacci et al., 2020; Meir and Barkan, 2020; Nicola et al., 2023).

## 3 Quantitative evaluation of the activity of new compounds in MAB-infected mice

The natural resistance of MAB to several drugs makes drug discovery difficult, as shown by hit rates in primary screens of less than 0.1% (Wu et al., 2018). Currently, drugs targeting protein synthesis (CLR/AZT, AMK, LNZ, and TGC) and peptidoglycan (PPG) synthesis (IMP and CFX) have been the mainstays of MAB treatment (Daley et al., 2020; Griffith and Daley, 2022).

Here, the differences reported in the literature between the colony forming units (CFUs) reductions in the lungs of MAB-infected mice after treatment with new compounds, or with positive control comparator drugs are shown in Table 1. Although the mouse models used were different, data were grouped on the basis of structure or function inhibited. Drugs were considered bactericidal when they caused ≥1 log_10_ reduction in lung CFU compared to CFU prior to treatment initiation (day 0 CFU) (Dartois and Dick, 2024b; Li et al., 2024; Xie et al., 2023), and bacteriostatic when reduction was <1 log_10_. Out of 17 compounds examined, 15 were bactericidal while the day 0 CFU data were not reported for 2 compounds. Chemical structure of the new compounds listed in Table 1 are shown in the Supplementary Figure 1.

**TABLE 1:** Colony forming unit reductions in the lungs of MAB-infected mice after treatment with new compounds, or with comparator drugs.

Structure or function inhibited	Compound	Target	Mice,^a^ route of infection^b^	mg of drug/kg, route of admini- stration^c^	Days of treatment	Bacteri- cidal (C) activity^d^	Log_10_ CFU reduction by the compound, compared to untreated mice	Log_10_ CFU reduction by the comparator drug, compared to untreated mice^e^									Δ CFU reduction, compared to untreated mice^f^	Refer-ence/s
								CLR	AZT	AMK	CLF	CFX	IMP	LNZ	BDQ	MXF		
Cell envelope	eAmSPC 2593	Efflux pump	SCID, IV	50, SC	8	C	>3.1			0.9							>2.2	Phelps et al., 2024
	eAmSPC 2593	Efflux pump	SCID, IV	30, SC	28	C	2.7			1.9							0.8	Phelps et al., 2024
	PBTZ169	DprE1	DI-BALB/c, IV	25, OR	28	C	3.33				2.3				3.09	2.25	1.03; 0.24; 1.08	Zheng et al., 2023
	COE-PNH2	Mycomem- brane	DI-C3, IN	5, IT	12	C	2.5			2.1							0.4	Zhang et al., 2024
	IC-25	MmpL3	SCID, IV	300, OR	9	C	1.83			1.89							∼ 0	Pandya et al., 2019
DNA replication	SPR720	DNA gyrase B	SCID, IV	100, OR	32	C	3.84	2.09		2.02	1.47						1.75; 1.82; 2.37	Cotroneo et al., 2024; Madani et al., 2022
	SPR720	DNA gyrase B	SCID, IV	50, OR	32	C	3.05	2.09		2.02	1.47						0.96; 1.03; 1.58	Cotroneo et al., 2024; Madani et al., 2022
	TPP8	DNA gyrase B	SCID, IN	25, IP	10	C	1.3	1.3								1	0; 0.3	Madani et al., 2022
	CGM	DNA sliding clamp	SCID, IN	250, OR	10	C	1.4	0.7									0.7	Aragaw et al., 2022
	EC/11716	DNA gyrase B	SCID, IN	400, OR	10	C	1.1	1.1								0.7	0; 0.4	Ganapathy et al., 2021
Protein synthesis	EC/11770	LeuRS	SCID, IN	10, OR	10	C	2	1.4									0.6	Ganapathy et al., 2023
	Epetraborole	LeuRS	DI-C3, AE	100, OR	28	C	4						3.4				0.6	Rimal et al., 2024a
	MRX-6038	LeuRS	CI-BALB/c, IN	10, SC	14	C	5	5						4.6			0; 0.4	Wu et al., 2022
	LCB01-0371	50S ribosomal subunit	C57BL/6, IN	100, OR	4	n.a.	0.5	0.2						0.5			0.3; 0	Kim T. S. et al., 2017
Energy metabolism	TBAJ-876	ATP synthase	SCID, IN	30, OR	10	C	2.2	1.4							1.3		0.8; 0.9	Sarathy et al., 2020a
	TBAJ-876	ATP synthase	SCID, IN	10, OR	10	C	1.7	1.4							1.3		0.3; 0.4	Sarathy et al., 2020a
	WX-081	ATP synthase	DI-BALB/c, IV	25, OR	28	C	3.2								3.2		0	Zheng et al., 2024
Other mechanism	MD3	Multiple mechanisms of action	SCID, IT	10, IT	8	n.a.	3			1.7							1.3	McDonald et al., 2024
	AR-12	Unknown	CI-BALB/c, IN	50, OR	14	C	3.6			3							0.6	Zhang et al., 2020
	OZ439	DosS	SCID, IV	50, OR	28	C	4.2		3.7	3.7	3.7		3.3				0.5; 0.5; 0.5; 0.9	Belardinelli et al., 2022

^a^SCID, severe combined immunodeficiency disease; DI-BALB/c, dexamethasone-immunosuppressed BALB/c; DI-C3, dexamethasone-immunosuppressed C3HeB/FeJ; CI-BALB/c, cyclophosphamide-immunosuppressed BALB/c. ^b^Route of infection: IV, intravenous; IN, intranasal; AE, aerosol; IT, intratracheal. ^c^Route of administration: SC, subcutaneous; OR, oral; IT, intratracheal; IP, intraperitoneal. ^d^≥1 log_10_ reduction in lung CFU compared to day 0 CFU (Dartois and Dick, 2024b; Li et al., 2024; Xie et al., 2023). n.a., not applied. ^e^CLR, clarithromycin; AZT, azithromycin; AMK, amikacin; CLF, clofazimine; CFX, cefoxitin; IMP, imipenem; LNZ, linezolid; BDQ, bedaquiline; MXF, moxifloxacin. ^f^Log_10_ CFU reduction by the compound – log_10_ CFU reduction by the comparator drug.

### 3.1 Cell envelope inhibitors

The mycobacterial cell envelope consists of three layers, including (i) cytoplasmic membrane, (ii) cell wall, and (iii) outer layer (Dulberger et al., 2020). The mycobacterial cell wall contains PPG covalently linked to arabinogalactan. The outer layer (myco-membrane) contains lipids and secreted proteins. In MTB, its outer leaflet is mostly composed of mycolic acids, which can be free or attached to trehalose sugar to make trehalose mono-mycolates (TMM) or trehalose di-mycolates. The inner leaflet contains mycolic acids covalently attached to the underlying arabinogalactan of the arabinogalactan-PPG matrix.

#### 3.1.1 Cytoplasmic membrane inhibitors: eAmSPC2593

An active area of research focused on development of inhibitors targeting efflux pumps. A recent report showed that spectinomycin (SPC) resistance in MAB was mediated by the expression of a TetV-like efflux pump (MAB2780c) (Parmar and Tocheva, 2023; Nguyen et al., 2024; Hurst-Hess et al., 2023). A subclass of spectinomycins (eAmSPC) was active against MAB in acute and chronic SCID mice infection models and synergized with anti-MAB antibiotics (Phelps et al., 2024). Strikingly, 8-days treatment with 50 mg/kg of the compound eAmSPC2593 completely cleared the infection below the limit of detection (>2.2-log_10_ CFU reduction) in pulmonary tissue, in comparison with AMK control therapy (Phelps et al., 2024). In the chronic model, 28-days treatment with 30 mg/kg of eAmSPC2593 decreased CFU by about 0.8-log_10_ more than AMK.

#### 3.1.2 Cell wall inhibitors: PBTZ169

PBTZ169 (macozinone) inhibits the decaprenyl-phosphoryl-ribose 2′-epimerase (DprE1), an enzyme involved in the synthesis of MTB cell wall arabinan polymers (Makarov and Mikusova, 2020; Imran et al., 2022; Dash et al., 2024). Zheng et al. (2023) evaluated the activity of the compound PBTZ169 in MAB-infected dexamethasone-immunosuppressed BALB/c (DI-BALB/c) mice, in comparison with CLF, bedaquiline (BDQ), moxifloxacin (MXF), and pretomanid (PRT) (former PA-824), a new anti-TB drug. Twenty-eight-days treatment with PBTZ169 decreased CFU by 1.03-, 0.24-, and 1.08-log_10_ more than CLF, BDQ, and MXF, respectively (Zheng et al., 2023).

#### 3.1.3 Mycomembrane and cytoplasmic membrane inhibitors: COE-PNH_2_

The conjugated oligoelectrolytes (COEs) are synthetic drugs designed to prevent antimicrobial resistance through mechanisms of action different from those of traditional antibiotics. COEs insert into cell membranes and function as electron transporters (Heithoff et al., 2023). A screen of non-cytotoxic COEs identified a lead compound, COE-PNH_2_, which was bactericidal against AR, NR persisters, and intracellular MAB (Zhang et al., 2024). COE-PNH_2_ targets the integrity of the bacterial envelope, affecting the mycomembrane and the bioenergetic functionality of the cytoplasmic membrane. Twelve-days treatment of MAB-infected dexamethasone-immunosuppressed C3HeB/FeJ (DI-C3) mice with COE-PNH_2_ was well tolerated and decreased CFU by about 0.4-log_10_ more than AMK.

#### 3.1.4 TMM translocation inhibitors: IC-25

It is known that the mycobacterial membrane protein Large 3 (MmpL3) is a transporter required for translocation of intracellularly synthesized TMM to cell envelope acceptors, resulting in inhibition of the outer layer formation (Franz et al., 2017; Sethiya et al., 2020). The TMM transport is essential for the formation of the MAB cell envelope that functions as the main physical barrier to drug entry. Indole-2-carboxamides (ICs) were identified as novel chemical scaffolds showing promising preclinical results against MTB and NTM (Pandya et al., 2019). The compound IC-25 targeted the MAB cytoplasmic membrane transporter MmpL3, resulting in abolition of mycolic acids translocation from the cytoplasm to the periplasmic space (Pandya et al., 2019). Nine-days treatment of MAB-infected SCID mice with IC-25 reduced CFU to levels similar to those of AMK.

### 3.2 DNA replication inhibitors

An important, category of drugs includes those targeting DNA replication. The DNA-gyrase inhibitors fluoroquinolones are widely used for treatment of multidrug-resistant (MDR) TB, but are not recommended for therapy of MAB infections (Daley et al., 2020). However, other compounds with ability to inhibit the DNA replication machineries were developed, including those inhibiting the DNA gyrase B (Stokes et al., 2020; Ganapathy et al., 2021) and the DNA sliding clump (Aragaw et al., 2022). The DNA gyrase, a highly conserved topoisomerase II enzyme, is a hetero-tetramer comprised of gyrase A (GyrA) and gyrase B (GyrB) subunits (Stokes et al., 2020).

#### 3.2.1 GyrB subunit inhibitors: SPR720 and TPP8

SPR719, the active moiety of the benzimidazole prodrug SPR720, inhibits the ATPase subunits of GyrB, a mechanism distinct from that of fluoroquinolones (Cotroneo et al., 2024). SPR719 was active against MAC and MAB strains resistant to CLR or AMK. In the chronic model, 32-days treatment of MAB-infected SCID mice with 100 mg/kg of SPR720 decreased CFU by 1. 75-, 1. 82-, and 2.37-log_10_ more than CLR, AMK, and CLF, respectively (Cotroneo et al., 2024). Also, 32-days treatment with 50 mg/kg of SPR720 decreased CFU by 0. 96-, 1. 03-, and 1.58-log_10_ more than CLR, AMK, and CLF, respectively.

Tricyclic pyrrolopyrimidines (TPPs) are a new class of antibacterials inhibiting the DNA GyrB. TPP8 inhibited the DNA supercoiling activity of the recombinant MAB enzyme (Madani et al., 2022). Indeed, 10-days treatment of MAB-infected SCID mice with TPP8 decreased CFU to the same level of CLR and by about 0.3-log_10_ more than MXF.

#### 3.2.2 DNA sliding clump inhibitors: cyclohexyl-griselimycin

Griselimycins (GMs) are cyclic depsipeptides originally isolated from *Streptomyces* species (Aragaw et al., 2022). The cyclohexyl analog CGM showed oral bioavailability and efficacy in TB mouse models. Binding studies and structural analyses showed that GMs targeted mycobacterial DnaN (Aragaw et al., 2022; Kling et al., 2015). The gene *dnaN* encodes the DnaN protein, that is crucial for DNA replication and repair, acting as a protein-protein interaction hub. DnaN protein partners interact with a specific hydrophobic cleft on the DnaN sliding clamp. Griselimycins bind to the same cleft, disrupting DnaN protein-protein interactions. Ten-days treatment of MAB-infected SCID mice with CGM decreased the CFU by about 0.7-log_10_ more than CLR (Aragaw et al., 2022).

#### 3.2.3 Novel bacterial topoisomerase inhibitors: EC/11716

Compared to fluoroquinolones, the novel bacterial topoisomerase inhibitors (NBTIs) are a new generation of compounds consisting of left- and right-hand portions linked together by a central unit (Ganapathy et al., 2021). *M. tuberculosis* gyrase inhibitors (MGIs) are a subclass of NBTIs. Ganapathy et al. (2021) selected EC/11716, a lead MGI with attractive TB activity, and profiled the compound for anti-NTM activity. Ten-days treatment of MAB-infected SCID mice with EC/11716 reduced CFU to the same level of CLR and by about 0.4-log_10_ more than MXF.

### 3.3 Protein synthesis inhibitors

Protein synthesis inhibitors active against MAB include the macrolides CLR and AZT (binding to the exit tunnel in the 50S ribosomal subunit), the aminoglycoside AMK (targeting the 30S ribosomal subunit), the tetracyclines TGC and omadacycline (OMD) (binding to the 30S subunit) and the oxazolidinone LNZ (inhibiting the peptidyl transferase activity of the 23S rRNA in the 50S subunit) (Wu et al., 2018; Daley et al., 2020; Clinical and Laboratory Standards Institute [CLSI], 2018; Dartois and Dick, 2024a; Egorova et al., 2021; Cocorullo et al., 2023). However, other drugs have been developed for ability to inhibit protein synthesis machineries like, for instance, those inhibiting the leucyl-tRNA synthetase (LeuRS) (Šlechta et al., 2023) and delpazolid (LCB01-0371), a new oxazolidinone (Kim et al., 2022).

#### 3.3.1 LeuRS inhibitors: benzoxaborole EC/11770, epetraborole, and MRX-6038

Aminoacyl-tRNA synthetases (aaRSs) are enzymes that attach each amino acid to its cognate tRNA molecule, enabling proper translation of the genetic code (Ganapathy et al., 2023). There are structural differences between the aaRS catalytic domains of prokaryotes and eukaryotes. Therefore, pathogen-specific aaRS inhibitors that did not target the human host enzymes were developed.

Given the antitubercular activity of the benzoxaborole GSK656, Ganapathy et al found that this compound and its close analog EC/11770 displayed anti-NTM activity and inhibited the MAB LeuRS (Ganapathy et al., 2023). Ten-days treatment of MAB-infected SCID mice with EC/11770 decreased CFU by about 0.6-log_10_ more than CLR.

The non-halogenated benzoxaborole epetraborole (EPT) was active against MAB *in vitro* and in mice (Rimal et al., 2024a). Twenty-eight-days treatment of MAB-infected DI-C3 mice with EPT decreased CFU by about 0.6-log_10_ more than IMP.

MRX-6038 is a novel LeuRS inhibitor with *in vivo* anti-MAB activity (Wu et al., 2022). Indeed, 14-days treatment of MAB-infected CI-BALB/c mice with MRX-6038 decreased CFU to the same level of CLR and by about 0.4-log_10_ more than LNZ.

#### 3.3.2 New oxazolidinones: LCB01-0371

LCB01-0371 (delpazolid, DLP) is an oxazolidinone with cyclic amidrazone (Kim et al., 2022; Kim T. S. et al., 2017). Four-days treatment of MAB-infected C57BL/6 mice with DLP decreased CFU to the same level of LNZ, and by about 0.3-log_10_ more than CLR (Kim T. S. et al., 2017). The phase 2a study NCT06004037 with DLP in patients with refractory *M. abscessus* pulmonary diseases (Dartois and Dick, 2024b) is ongoing.

### 3.4 Energy metabolism inhibitors

Energy metabolism in mycobacteria, in particular the oxidative phosphorylation pathway, has emerged as an object of intense microbiological investigation and as a novel target pathway in drug discovery (Egorova et al., 2021; Bald et al., 2017). During electron transport along the respiratory chain, protons are pumped across the membrane, leading to a proton motive force used by ATP synthase for ATP synthesis.

#### 3.4.1 ATP synthase inhibitors: TBAJ-876 and WX081

Bedaquiline is a diarylquinoline used for treatment of MDR-TB. It inhibits the ATP synthase through targeting the c and ε subunits (Sarathy et al., 2020b). Bedaquiline also targets the MAB ATP synthase (Dupont et al., 2017) and inhibits MAB clinical isolates *in vitro*, with bacteriostatic and bactericidal activity against AR and NR cells, respectively (Le Moigne et al., 2020; Martins et al., 2021).

Recent medicinal chemistry campaigns resulted in the discovery of 3,5-dialkoxypyridine analogues of BDQ which were less lipophilic and had higher clearance and lower cardiotoxic potential than it. One of this compounds, TBAJ-876, showed *in vivo* activities against MAB, similar to its BDQ parent (Sarathy et al., 2020a). Indeed, 10-days treatment of MAB-infected SCID mice with 30 mg/kg of TBAJ-876 decreased CFU by about 0.8- and 0.9-log_10_ more than CLR and BDQ, respectively. When TBAJ-876 was used at 10 mg/kg, CFU decreased by about 0.3- and 0.4-log_10_ more than CLR and BDQ, respectively.

Finally, the sudapyridine (WX-081), another structural analog of BDQ, showed anti-TB and -NTM activity but, unlike BDQ, did not prolong the QT interval in animal models (Zheng et al., 2024). Twenty-eight-days treatment of MAB-infected DI-BALB/c mice with WX-081 decreased CFU to the same level of BDQ.

### 3.5 Compounds with other mechanisms of inhibition: MD3, AR-12, and OZ439

#### 3.5.1 MD3, a nitric-oxide releasing prodrug

Nitric oxide kills bacteria by inducing oxidative and nitrosative stresses, damaging bacterial cell membranes via lipid peroxidation, altering protein activity via protein nitrosation, and inducing DNA and RNA damage via reactive nitrogen species (McDonald et al., 2024). The anti-MAB activity of the bactericidal NO-releasing prodrug methyl tris diazeniumdiolate (MD3) was evaluated *in vitro* and *in vivo*. Eight-days treatment of MAB-infected SCID mice with MD3 decreased CFU by about 1.3-log_10_ more than AMK (McDonald et al., 2024).

#### 3.5.2 AR-12, a celecoxib derivative

In humans, AR-12 (OSU-03012), a novel celecoxib derivative lacking the cyclooxygenase-2 inhibitor activity, repressed host cell chaperone machinery, inhibited kinase pathways and upregulated autophagy (Zhang et al., 2020). Several studies indicated that AR-12 exerted biological effects also against fungi, bacteria, parasites and viruses (Zhang et al., 2020; Collier et al., 2016; Chan et al., 2018; Krishnamurthy et al., 2019). AR-12 was active also against MAB, but no information is known on its mechanism/s of action. Fourteen-days treatment of MAB-infected CI-BALB/c mice with AR-12 decreased CFU by about 0.6-log_10_ more than AMK (Zhang et al., 2020).

#### 3.5.3 OZ439, a DosS inhibitor

Non-tuberculous mycobacteria and MTB share the ability to persist within granulomatous structures in a NR state, contributing to drug tolerance and treatment failure in chronically infected individuals (Belardinelli et al., 2022). *M. tuberculosis* survives this stress by inducing a dormancy regulon under control of the three-component regulatory system, DosRST. Inhibitors of the response regulator DosR and of the two sensor histidine kinases, DosS and DosT, are being sought for their potential to shorten TB treatment. *M. abscessus* is endowed with a DosRS system, although it lacks the sensor histidine kinase, DosT. The MAB DosRS two-component system controls a species-specific regulon required for adaptation to hypoxia (Simcox et al., 2023). Genetic disruption of *dosRS* impairs MAB adaptation to hypoxia, resulting in decreased survival after oxygen depletion, and inhibition of biofilm formation (Belardinelli et al., 2022). Antimalarial drugs or drug candidates, artemisinin, OZ277, and OZ439 (artefenomel), can target the MAB DosS-mediated hypoxic signaling. OZ439 showed bactericidal activity comparable to standard-of-care antibiotics in chronically infected mice (Belardinelli et al., 2022). Indeed, 28-days treatment of MAB-infected SCID mice with OZ439 decreased CFU by about 0.5-log_10_ more than AZT, AMK, and CLF and by about 0.9-log_10_ more than IMP.

## 4 Quantitative evaluation of the activity of drug combinations in MAB-infected mice

With cure rates below 50%, there is an urgent need for drug combinations that can shorten MAB therapies.

Colony forming unit reductions by drug combinations compared to day 0 CFU and to untreated mice at the end of treatment are shown in Tables 2, 3, respectively. Table 2 showed that out of 14 combinations examined, 12 were bactericidal (≥1 log_10_ CFU reduction), while for 2 of them no data were reported (combinations 6–7). Epetraborole, OMD, BDQ, CLF, and LNZ-CLF were bacteriostatic (<1 log_10_ CFU reduction), while all other compounds/combination components were bactericidal. The comparator drugs CLR and RFB were low-level bactericidal agents (1.1–1.37 log_10_ CFU reduction).

**TABLE 2:** Colony forming unit reductions by drug combinations in the lungs of MAB-infected mice, compared to CFU prior to treatment initiation (day 0 CFU).

Structure/s or function/s inhibited	Combination^a^	Target/s			Mice^b^	mg of drug/kg	Days of treatment	Log_10_ CFU reduction compared to day 0 CFU (bactericidal, C, ≥1; bacteriostatic, S, <1)					
								Combination	IMP	DRP	BPN	CFX	EPT
Cell envelope	1. IMP-DRP	Peptidoglycan (PPG)			DI-C3	100-200	28	2.55 (C)	2.13 (C)	1.46 (C)			
	2. IMP-BPN	PPG			DI-C3	100-200	28	3.05 (C)	2.13 (C)		2.33 (C)		
	3. IMP-CFX	PPG			DI-C3	100-200	28	3.02 (C)	2.13 (C)			n.a.	
	4. BPN-AVB	PPG			DI-C3	200-64	28	2.96 (C)			2.33 (C)		
	5. TBP-AVB	PPG			SCID	400-200	10	1.2 (C)					
	6. CFX-ORT	PPG			DI-BALB/c	200-50	10	n.a.					
	7. MRP-ORT	PPG			DI-BALB/c	200-50	10	n.a.					
PPG + protein or ATP synthesis	8. BPN-OMD	PPG + 30S ribosomal subunit			DI-C3	200-15	28	2.0 (C)			2.0 (C)		
	9. IMP-OMD-AMK	PPG + 30S + 30S			DI-C3	100-15-150	28	2.4 (C)					
	10. IMP-BDQ	PPG + ATP synthase			C3HeB/FeJ	100-30	13	1.2^c^ (C)					
Other combinations	11. EPT-NRV	LeuRS			SCID	10-3.3	10	1.9 (C)					0.9 (S)
	12. OMD-LNZ-CLF	30S + 50S + cell membrane?			DI-C3	15-100-25	42	1.3 (C)					
	13. CLR-AMK-SPR720	50S + 30S + gyrB			SCID	250-150-50	32	3.93 (C)					
	14. CLR-AMK-CLF-SPR720	50S + 30S + cell membrane? + gyrB			SCID	250-150-20-50	32	3.97 (C)					
**Structure/s or function/s inhibited**	**Combination^a^**	**Log_10_ CFU reduction compared to day 0 CFU (bactericidal, C, ≥1; bacteriostatic, S, <1)**											**Reference/s**
		**OMD**	**BDQ**	**SPR720**	**CLF**	**AMK**	**IMP-AMK**	**LNZ-CLF**	**CLR-AMK**	**CLR-AMK-CLF**	**CLR**	**Rifabutin**	
Cell envelope	1. IMP-DRP												Story-Roller et al., 2019b
	2. IMP-BPN												Story-Roller et al., 2019b
	3. IMP-CFX												Story-Roller et al., 2019b
	4. BPN-AVB												Story-Roller et al., 2019b
	5. TBP-AVB										1.2 (C)		Negatu et al., 2023
	6. CFX-ORT												Wang et al., 2021
	7. MRP-ORT												Wang et al., 2021
PPG + protein or ATP synthesis	8. BPN-OMD	0.9 (S)											Rimal et al., 2023
	9. IMP-OMD-AMK	0.6 (S)					1.9 (C)						Rimal et al., 2024b
	10. IMP-BDQ		0.8 (S)										Le Moigne et al., 2020
Other combinations	11. EPT-NRV											1.1 (C)	Sullivan et al., 2021
	12. OMD-LNZ-CLF	0.8 (S)						0.5 (S)					Ignatius et al., 2024
	13. CLR-AMK-SPR720			2.33 (C)		1.3 (C)			3.27 (C)				Cotroneo et al., 2024
	14. CLR-AMK-CLF-SPR720			2.33 (C)	0.75 (S)	1.3 (C)				2.13 (C)	1.37 (C)		Cotroneo et al., 2024

^a^IMP, imipenem; DRP, doripenem,; BPN, biapenem; CFX, cefoxitin; AVB, avibactam; TBP, tebipenem; ORT, oritavancin; MRP, meropenem; OMD, omadacycline; AMK, amikacin; BDQ, bedaquiline; EPT, epetraborole; NRV, norvaline; LNZ, linezolid; CLF, clofazimine; CLR, clarithromycin. ^b^Dexamethasone-immunosuppressed C3HeB/FeJ, DI-C3; dexamethasone-immunosuppressed BALB/c, DI-BALB/c; severe combined immunodeficiency disease, SCID. ^c^Refers to CFU of drug untreated mice on day 13 (Le Moigne et al., 2020).

**TABLE 3:** Colony forming unit reductions by drug combinations in the lungs of MAB-infected mice, compared to untreated mice at the end of treatment.

Structure/s or function/s inhibited	Combi- nation^a^	Route of admini- stration^b^	Log_10_ CFU reduction by drug combination at the end of treatment	Log_10_ CFU reduction by drug combination component/s and comparator drugs at the end of treatment															Δ log_10_ CFU reduction at the end of treatment^c^	Reference
				IMP	MRP	DRP	BPN	CFX	ORT	EPT	OMD	BDQ	IMP- AMK	LNZ- CLF	CLR- AMK	CLR- AMK- CLF	CLR	Rifa- butin		
Cell envelope	1. IMP-DRP	P-P	6.17	5.75		5.08													0.42; 1.09	Story-Roller et al., 2019b
	2. IMP-BPN	P-P	6.67	5.75			5.95												0.92; 0.72	Story-Roller et al., 2019b
	3. IMP-CFX	P-P	6.64	5.75				n.a.											0.89	Story-Roller et al., 2019b
	4. BPN-AVB	P-P	6.58				5.95												0.63	Story-Roller et al., 2019b
	5. TBP-AVB	O-O^d^	1.1														**1.1**		0	Negatu et al., 2023
	6. CFX-ORT	P-P	2.3					0.4	0.4								**1.1**		1.9; 1.9; 1.2	Wang et al., 2021
	7. MRP-ORT	P-P	2.3		0.4				0.4								**1.1**		1.9; 1.9; 1.2	Wang et al., 2021
PPG + protein or ATP synthesis	8. BPN-OMD	P-P*^e^*	2.9				2.9				1.8								0; 1.1	Rimal et al., 2023
	9. IMP-OMD-AMK	P-P*^e^*-P	4.8								3		4.3						1.8; 0.5	Rimal et al., 2024b
	10. IMP-BDQ	P-O	1.2	0								0.8							1.2; 0.4	Le Moigne et al., 2020
Other combinations	11. EPT-NRV	O-O	1.8							0.9								1.0	0.9; 0.8	Sullivan et al., 2021
	12. OMD-LNZ- CLF	P*^e^*-O-O	4.6								4.1			3.8					0.5; 0.8	Ignatius et al., 2024
	13. CLR-AMK- SPR720	O-P-O	4.35												2.56				1.79	Cotroneo et al., 2024
	14. CLR-AMK-CLF- SPR720	O-P-O-O	4.69													2.85			1.84	Cotroneo et al., 2024

^a^IMP, imipenem; DRP, doripenem; BPN, biapenem; CFX, cefoxitin; AVB, avibactam; TBP, tebipenem; ORT, oritavancin; MRP, meropenem; OMD, omadacycline; AMK, amikacin; BDQ, bedaquiline; EPT, epetraborole; NRV, norvaline; LNZ, linezolid; CLF, clofazimine; CLR, clarithromycin. ^b^P, parenteral; O, oral. ^c^Log_10_ CFU reduction by the drug combination – log_10_ CFU reduction by the drug combination component/s, or the comparator drugs. ^d^Administered as TBP-pivoxil plus AVB-ARX 1796 oral prodrugs, respectively (Negatu et al., 2023). ^e^OMD lacks oral bioavailability in mice. Therefore, a subcutaneous dose (15 mg/kg once daily) equivalent to 300 mg oral dose in humans was used (Rimal et al., 2023; Rimal et al., 2024b; Ignatius et al., 2024).

### 4.1 Cell envelope inhibitors: dual β-lactams, β-lactam-β-lactamase inhibitors, and β-lactam-lipoglycopeptides

Bactericidal antibiotics targeting the same cellular process often achieve killing when combined (Dartois and Dick, 2024b). One such process includes combinations of PPG synthesis inhibitors involving transpeptidases and carboxypeptidases, which are inhibited by β-lactam antibiotics. During the final steps of PPG synthesis, MAB utilizes two enzyme classes, the D,D-transpeptidases (DDTs, also known as penicillin-binding proteins) and the L,D-transpeptidases (LDTs), to generate 4→3 and 3→3 linkages between step peptides, respectively (Story-Roller et al., 2019a). It is known that MAB contains predominantly LDTs, but also DDTs and one carboxypeptidase (Pozuelo Torres and van Ingen, 2024). *M. abscessus* also contains a β-lactamase (Bla_MAB_) which inactivates several β-lactams.

#### 4.1.1 Dual β-lactams (combinations 1–3)

In MAB, several carbapenems [e.g., IMP, doripenem (DRP), biapenem (BPN), and meropenem (MRP)] inhibits LDTs, while cephalosporins (e.g., CFX) and penicillins inhibits DDTs (Story-Roller et al., 2019a). Several studies suggested that combinations of bactericidal agents including two β-lactam antibiotics (dual β-lactams) exhibited *in vitro* and *in vivo* synergy against MAB (Story-Roller et al., 2019a; Pozuelo Torres and van Ingen, 2024; Story-Roller et al., 2019b). MAB efficiently grew in DI-C3 mice (Story-Roller et al., 2019b) and 28-days treatment with IMP-DRP, IMP-BPN, and IMP-CFX, each containing two parenteral bactericidal β-lactams, exhibited strong synergy among them, as shown by 2.55–3.05 log_10_ CFU reduction (Table 2), and 6.17–6.67 log_10_ reduction (Table 3), respectively. Some support to the importance of dual β-lactams for treatment of MAB infections in humans was reported in various clinical studies in which patients were treated with IMP-amoxicillin (AMX) (Moguillansky et al., 2023), IPM-ceftaroline (CFT) (Alahmdi et al., 2023), and MRP-CFT (Becken et al., 2024). A commentary review on evidences and questions regarding dual β-lactam therapy for MAB infections was also reported (Lippincott and Lamichhane, 2024).

#### 4.1.2 β-Lactams-β-lactamase inhibitors (combinations 4–5)

The widely applied β-lactamase inhibitor (BLI) clavulanic acid did not bind to Bla_MAB_ (Pozuelo Torres and van Ingen, 2024). Conversely, the BLIs avibactam (AVB), relebactam (RLB), zidebactam, and nacubactam bound to Bla_MAB_ and increased the susceptibility to β-lactams (Pozuelo Torres and van Ingen, 2024; Misawa et al., 2022). Recent studies showed that the BLI durlobactam (DRL) behaved as a “dual action” compound by inactivating Bla_MAB_, LDTs and DDTs more potently than AVB (Dousa et al., 2022; Dousa et al., 2024; Shin et al., 2024; Sakoulas, 2025). Durlobactam, but not AVB, inhibited multiple components of MAB cell wall machinery at concentrations below clinically available levels and time-kill studies showed that DRL plus IMP or two other β-lactams achieved near complete MAB sterilization. In MAB-infected DI-C3 mice, 28-days treatment with the bactericidal BPN plus AVB (BPN-AVB) caused 2.96-log_10_ CFU reduction (Table 2), and 6.58-log_10_ reduction (Table 3), respectively (Story-Roller et al., 2019b). Tebipenem (TBP) plus AVB (TBP-AVB) was also promising *in vitro* and *in vivo* (Pozuelo Torres and van Ingen, 2024; Negatu et al., 2022; Fröberg et al., 2023; Negatu et al., 2023). MAB did not replicate in SCID mice and 10-days treatment with oral TBP-AVB (administered as TBP-pivoxil plus AVB-ARX 1796 prodrugs, respectively), decreased CFU to the same levels of oral CLR (Tables 2, 3; Negatu et al., 2023). In humans, IMP-cilastatin-RLB-AMX was well tolerated and patients improved clinically and radiologically after the first phase of treatment (Vogiatzoglou et al., 2024). β-Lactam therapy with MRP, ceftazidime-AVB and two phages also provided clinical improvement (Cristinziano et al., 2024).

#### 4.1.3 β-Lactams-lipoglycopeptides (combinations 6–7)

Oritavancin (ORT) is a bactericidal lipoglycopeptide that induces cell membrane damages and blocks transglycosylation and transpeptidation steps during PPG biosynthesis (Zhanel et al., 2012; Wang et al., 2021). Ten-day treatment of DI-BALB/c mice with CFX and MRP (two bactericidal agents) plus ORT (CFX-ORT and MRP-ORT, respectively) reduced CFU by about 2.3-log_10_ compared to untreated mice (Table 3), consistent with the knowledge that ORT synergized with these and other drugs *in vitro* (Wang et al., 2021). No CFU data were reported on day 0.

### 4.2 Combinations of inhibitors of PPG plus protein or ATP synthesis

#### 4.2.1 BPN-OMD (combination 8)

One of the novel antibiotics being studied for treatment of MAB disease is OMD, a semisynthetic tetracycline that targets the 30S ribosomal subunit, essential for protein synthesis (Rimal et al., 2023). *M. abscessus* efficiently grew in DI-C3 mice and 28-days treatment with BPN-OMD (bactericidal and bacteriostatic drugs, respectively), reduced CFU by about 2-log_10_ (Table 2), and 2.9-log_10_ (Table 3), respectively. The phase 2-study NCT04922554 with oral OMD vs. placebo in patients with MAB-PD (Dartois and Dick, 2024b) has been completed.

#### 4.2.2 IMP-OMD-AMK (combination 9)

Twenty-eight-day treatment of MAB-infected DI-C3 mice with IMP-OMD-AMK (bactericidal, bacteriostatic and bactericidal drugs, respectively) reduced CFU by about 2.4-log_10_ (Table 2), and 4.8-log_10_ (Rimal et al., 2024b), respectively. These data were obtained by infecting mice with the MAB reference strain ATCC 19977. However, the same group of investigators found no major differences between IMP-AMK and IMP-OMD-AMK when mice were infected with clinical isolates (Ignatius et al., 2024).

#### 4.2.3 IMP-BDQ (combination 10)

Bedaquiline inhibits the MAB ATP synthase (Dupont et al., 2017). *In vitro*, BDQ was bacteriostatic against AR MAB and antagonized the bactericidal activity of IMP (Martins et al., 2021). Contrarily, BDQ was bactericidal against nutrient-starved NR persisters, while IMP was not, and BDQ drove the activity of the combination. In intravenously infected dexamethasone-untreated C3HeB/FeJ mice, MAB was consistently inhibited by the immune response after 13 days of treatment. IMP-BDQ further decreased CFU by 1.2 log_10_, mostly due to BDQ (0.8-log_10_, Table 3).

### 4.3 Other combinations

#### 4.3.1 EPT-norvaline (combination 11)

Epetraborole inhibits the MAB LeuRS, an enzyme essential in protein synthesis (Rimal et al., 2024a; Sullivan et al., 2021). Since EPT-resistant MAB mutants lost LeuRS editing activity, they became susceptible to misaminoacylation with leucine mimics like the non-proteinogenic amino acid norvaline (NRV), an isomer of valine (Sullivan et al., 2021). In SCID mice MAB did not replicate after infection, and treatment with EPT (a bacteriostatic agent) plus NRV (EPT-NRV) for 10 days decreased CFU by about 1.9-log_10_ (Table 2) and 1.8-log_10_ (Table 3), respectively.

#### 4.3.2 OMD-LNZ-CLF (combination 12)

*Mycobacterium abscessus* efficiently replicated in DI-C3 mice and 42-days treatment with OMD-LNZ-CLF (three bacteriostatic drugs) reduced CFU by about 1.3-log_10_ (Table 2), and 4.6-log_10_ (Table 3), respectively (Ignatius et al., 2024).

#### 4.3.3 CLR-AMK-SPR720 and CLR-AMK-CLF-SPR720 (combinations 13–14)

SPR719, the active moiety of SPR720, inhibits the ATPase subunits of the DNA gyrase B (Stokes et al., 2020). *M. abscessus* replicated in the chronic SCID mice infection model, and 32-days treatment with CLR-AMK-SPR720 and CLR-AMK-CLF-SPR720 decreased CFU by 3.93- and 3.97-log_10_ (Table 2) and 4.35- and 4.69-log_10_ (Table 3), respectively (Cotroneo et al., 2024). Clofazimine was bacteriostatic and SPR720 was bactericidal, as shown by 0.75- and 2.33-log_10_ CFU reduction, respectively, compared to day 0. In these combinations the comparator drug CLR was a low-level bactericidal agent (1.37-log_10_ CFU reduction) (Table 2).

## 5 Discussion

This review provides a comprehensive update on the activity of new and old compounds and of their combinations in MAB-infected mice, recently reported in the literature. While TB murine models have been important in predicting drug efficacy, preclinical evaluation of anti-MAB drugs is more challenging due to the difficulty of establishing a progressive and sustained pulmonary infection with this pathogen in mice (Dartois et al., 2024; Maggioncalda et al., 2020). To address these difficulties, three workshops were hosted in 2023 by the CF foundation and the National Institute of Allergy and Infectious Diseases to review the current murine models of MAB infections, including phenotypic distinctions among the various mouse strains and several other issues. The key points from these workshops were recently summarized and reviewed (Dartois et al., 2024).

Presently, models of 1–2 weeks acute infection and of chronic infection with stable bacterial burden for up to 56 days are used. Acute infection models are preferred to support early-stage drug discovery.

Table 1 shows efficacy of 17 new compounds in mice lungs. All these molecules showed levels of activity greater than or equal to those of comparator drugs, i.e., antibiotics recommended for treatment of MAB infections in clinical guidelines, namely CLR, AZT, AMK, CLF, CFX, IMP, and LNZ (Daley et al., 2020; Dartois et al., 2024; Griffith and Daley, 2022; Haworth et al., 2017), and of other drugs such as BDQ and MXF used as comparator drugs in three papers each (Zheng et al., 2023; Ganapathy et al., 2021; Madani et al., 2022; Sarathy et al., 2020a; Zheng et al., 2024). Two dosages were used for eAmSPC 2593, SPR720 and TBA-J876. The acute model (4–14 days of treatment) was preferred in most of efficacy testing procedures (13 out of 20 experimental protocols) while the chronic model (28–32 days of treatment) was used in seven protocols. *M. abscessus* is typically considered of low virulence, necessitating immunodeficient mouse models (Dartois et al., 2024). Indeed, in 19 out of 20 protocols the investigators used SCID, DI-BALB/c, DI-C3, and CI-BALB/c mice (13, 2, 2, and 2 protocols, respectively). Intranasal, intravenous, aerosol, and intratracheal infection routes (10, 8, 1, and 1, respectively) were used. Drugs were administered by oral, subcutaneous, intratracheal, and intraperitoneal routes (14, 3, 2, and 1, respectively). The strains used for infection were *M. abscessus abscessus* ATCC 19977, K21, 1513, CIP108297, and *M. abscessus bolletii* (9, 7, 2, 1, and 1, respectively).

Overall, 15 out of 17 compounds decreased CFU more efficiently than comparator drugs. Fifteen compounds were bactericidal (≥1 log_10_ CFU reduction compared to day 0) (Dartois and Dick, 2024b; Li et al., 2024; Xie et al., 2023); these values were not reported for LCB01-0371 and MD3. The compounds which decreased the CFUs by >1 log_10_ for at least 1 comparator drug were e-AmSPC 2593, PBTZ169, SPR720, and MD3, but only PBTZ169 and SPR720 were oral, i.e., the route preferred for longtime treatment of MAB infections.

PBTZ169 was bactericidal *in vivo* against MAB, *M. chelonae, M. fortuitum*, and bacteriostatic against *M. avium* (Zheng et al., 2023). It was 10-times less cytotoxic than the lead TB compound benzothiazinone 043 (BTZ043) from which it derived (Makarov et al., 2014). In MTB, PBTZ169 covalently repressed DprE1, involved in the biosynthesis of cell wall components with synergic impacts with BDQ in preclinical models (Gawad and Bonde, 2018).

SPR720 (fobrepodacin), a bioavailable ester prodrug of SPR719, inhibits the ATPase subunits of DNA gyrase B, a target not exploited by currently used antibiotics, and therefore, no cross-resistance is expected to such agents (Winthrop et al., 2023). SPR720 is generally well tolerated, with mainly gastrointestinal and headache adverse events of mild or moderate severity. SPR720 just completed phase 2 dose-ranging studies (NCT05496374 and NCT04553406) to evaluate efficacy, safety, tolerability, and pharmacokinetic (PK) vs. placebo in MAC pulmonary disease (Dartois and Dick, 2024a; Cotroneo et al., 2024).

As to the combinations, in order to find a common thread that somehow connects their killing activities, we considered the recent proposal of Dartois and Dick (2024b) based on the prioritization of approved oral and bactericidal drug classes. Briefly, they suggested to target vital MAB processes with combinations of oral and bactericidal antibiotics that are part of current treatment guidelines. In particular, they proposed a model whereby the combination β-lactam-fluoroquinolone-rifamycin causes irreversible damages (cell lysis by β-lactam and DNA fragmentation by fluoroquinolone) that activate transcriptional stress responses aiming at damage control which, in turn, are inhibited by rifamycin RNA polymerase inhibitors. Detrimental proteome imbalances and damage repair will dysregulate the metabolism and generates reactive oxygen species as byproducts, causing additional damages to macromolecules, homeostatic systems collapse, and cell death.

In the papers examined here, no combination contained these three bactericidal drugs. However, judging by the CFU reductions shown in Tables 2, 3, in the chronic model of infection (≥28 days) the most potent combinations were combinations 1–4, containing injectable bactericidal agents including two β-lactams (IMP-DRP, IMP-BPN, and IMP-CFX), and a β-lactam plus a β-lactamase inhibitor (BPN-AVB). These combinations were highly bactericidal (from 2.55- to 3.05-log_10_ CFU reduction compared to day 0 CFU) and highly effective (from 6.17- to 6.67-log_10_ CFU reduction at the end of treatment) (Story-Roller et al., 2019b). These observations demonstrated synergistic activities between combination components that contributed to CFU reduction both on day 0 and at the end of treatment. Dual β-lactam therapy offers a promising approach to the treatment of MAB infections, especially when macrolide resistance is present (Moguillansky et al., 2023; Alahmdi et al., 2023; Becken et al., 2024; Lippincott and Lamichhane, 2024; Vogiatzoglou et al., 2024; Cristinziano et al., 2024). Although clinical efficacy of β-lactam combinations in humans is encouraging, it is important to consider concerns about toxicity, particularly when longer treatment courses are used with some carbapenems, as they may cause seizures and other neurotoxic effects (Grill and Maganti, 2011). Further research is needed to evaluate the efficacy and safety of dual β-lactam therapy for MAB infections, including optimal combinations of compounds and potential strategies to minimize adverse effects.

Among the other combinations, the activity of the bactericidal compounds was usually slightly increased by bacteriostatic agents. For instance, addition of the oral bacteriostatic OMD to BPN (combination 8) did not increase the activity of BPN compared to CFU on day 0 (2-log_10_ reduction by both BPN and BPN-OMD, Table 2) and at the end of treatment (2.9-log_10_ reduction by both BPN and BPN-OMD on day 28, Table 3; Rimal et al., 2023). Similarly, addition of OMD to the bactericidal IMP and AMK (combination 9) slightly increased the activity of IMP-AMK compared to day 0 CFU (from 1.9-log_10_ reduction by IMP-AMK to 2.4-log_10_ reduction by IMP-OMD-AMK, Table 2) and at the end of treatment (from 4.3-log_10_ reduction by IMP-AMK to 4.8-log_10_ reduction by IMP-OMD-AMK on day 28, Table 3; Rimal et al., 2024b).

Also, addition of the oral bacteriostatic CLF to the combination 13 (CLR-AMK-SPR720, formed by 3 bactericidal drugs) slightly increased the activity of the combination 14 (CLR-AMK-CLF-SPR720), compared to day 0 CFU (from 3.93-log_10_ reduction by CLR-AMK-SPR720 to 3.97-log_10_ reduction by CLR-AMK-CLF-SPR720, Table 2) and at the end of treatment (from 4.35-log_10_ reduction by CLR-AMK-SPR720 to 4.69-log_10_ reduction by CLR-AMK-CLF-SPR720, on day 32, Table 3; Cotroneo et al., 2024).

However, sometimes the effect of the bacteriostatic drugs in the combinations was difficult to explain, as in the case of the combination 12 (OMD-LNZ-CLF, formed by three oral bacteriostatic agents) that, was low effective in comparison to day 0 CFU (1.3-log_10_ CFU reduction by OMD-LNZ-CLF, Table 2), but highly active at the end of treatment (4.6-log_10_ CFU reduction by OMD-LNZ-CLF on day 42, Table 3; Ignatius et al., 2024). This was likely due to a late synergism between the three agents, which inhibited protein synthesis (OMD and LNZ) and, probably, cell membrane functions (CLF) (Cocorullo et al., 2023).

Also, it is difficult to explain the role of the combination components in the acute mice infection models (10–13 days). For instance, as to the combination 5 (oral TBP-AVB), CFU reductions by TBP alone compared to day 0 and at the end of treatment were not reported (Negatu et al., 2023).

Furthermore, as to the injectable combinations 6 (CFX-ORT) and 7 (MRP-ORT), no data were reported on their activity in comparison to day 0 CFU (Maggioncalda et al., 2020). However, it is known that, *in vitro*, ORT was bactericidal and able to synergize with various drugs (Wang et al., 2021). These properties may explain the quite good activity of CFX-ORT and MRP-ORT at the end of treatment (2.3-log_10_ CFU reduction by both combinations on day 10, Table 3).

As to the combination 11 (EPT-NRV), EPT was bacteriostatic in mice (Table 2). Therefore, increase in activity compared to day 0 (from 0.9-log_10_ CFU reduction by EPT to 1.8-log_10_ reduction by EPT-NRV) and at the end of treatment (from 0.9-log_10_ reduction by EPT to 1.9-log_10_ reduction by EPT-NRV on day 10, Table 3), was due to NRV (Sullivan et al., 2021).

Finally, as to the combination 10 (IMP-BDQ), the immune response developed in intravenously infected dexamethasone-untreated C3HeB/FeJ mice inhibited MAB growth by 2.7-log_10_ CFU, compared to day 0 (Le Moigne et al., 2020). Thirteen-days treatment with IMP-BDQ further decreased the initial MAB burden by 1.2-log_10_ CFU, with BDQ contributing to a 0.8-log_10_ reduction (Table 3).

In all these investigations, PK studies conducted in satellite mice, including blood drug concentrations and many other PK parameters, have been reported or cited in previous publications to optimize treatment regimens and establish tolerability. This was essential for a complete understanding of safety profiles and therapeutic indices, guiding further drug development and risk-benefit assessment.

Overall, this review demonstrated that no combination killed all the MAB cells in the lungs of mice, which contain both AR and NR, drug-tolerant bacilli (persisters). As with TB, persisters are a major cause of long treatment periods for MAB infections. Based on CFU decrease, chronic infection models, in which the majority of bacteria are in the NR state, provided more information than acute models to evaluate the bactericidal activity of the combinations. However, since MAB infections in humans last for years or a lifetime, it would be important to use chronic infection models with longer durations than currently used, to better evaluate drug activity against NR bacilli.

## 6 Conclusion

*Mycobacterium abscessus* treatment is complicated by the lack of bactericidal potency of antibiotics, the ability of the microorganism to persist in the hypoxic microenvironment of granulomas, the intrinsic drug resistance. Therefore, there is an urgent need to find oral and bactericidal drugs to treat pulmonary and extrapulmonary MAB disease.

In general, in this review the activity of the bactericidal compounds was only slightly augmented by the bacteriostatic agents. Instead, the combinations formed by bactericidal agents such as dual β-lactams and/or a β-lactam plus a β-lactamase inhibitor were the most active after 28 days of treatment, with >6-log_10_ CFU reduction compared to untreated mice, but they were all administered by the parenteral route. Attempts to find oral β-lactams with strong bactericidal synergies were reported, including TBP, sulopenem, and cefuroxime combined with AMX in the presence of AVB (Negatu et al., 2022). Recently, the oral triple-drug combination containing TBP-AVB, MXF, and RFB was shown to be bactericidal *in vitro* against MAB AR and NR cells, and effective in an acute mouse model of infection after 7 days of treatment (Sarathy et al., 2025).

Further research is needed to identify other synergistic combinations effective not only against currently used MAB reference strains, but also against clinical isolates and MAB subspecies, making it difficult to develop universally effective treatments. More standardized and validated mouse models are necessary to improve reproducibility and accelerate translation into clinical trials. Trials in MAB disease are urgently needed, and dual β-lactams and/or β-lactams plus β-lactamase inhibitors appear to be prime candidates for these studies.

Overall, the data obtained from the articles examined so far indicated that the studies using MAB-infected mice models have been very useful for evaluating drug treatments and that it is necessary and urgent to continue systematic *in vivo* studies on the activity of combinations containing bactericidal agents such as repurposed drugs, currently used drugs or new, preferably oral, compounds, to eradicate NR MAB persisters at the disease sites.
