# Supplementary material for: Activity of combinations of bactericidal and bacteriostatic compounds in Mycobacterium abscessus-infected mice: an overview
Source: Front Microbiol. 2025 Aug 1;16:1616149. doi: 10.3389/fmicb.2025.1616149 (PMC12354516; doi:10.3389/fmicb.2025.1616149)

Supplementary Figure 1. Chemical structure of the new compounds listed in the table 1

Cell envelope

eAmSPC2593

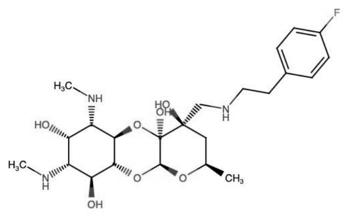

PBTZ169

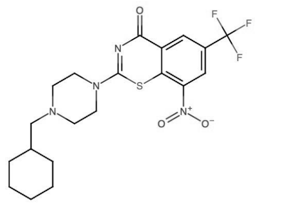

COE-PNH$_2$

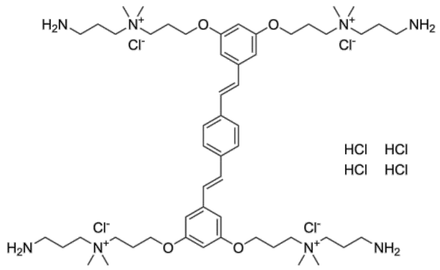

IC-25

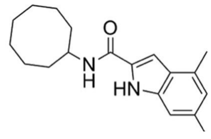

DNA replication

SPR720

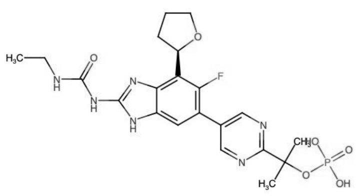

TPP8

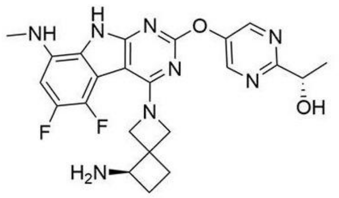

CGM

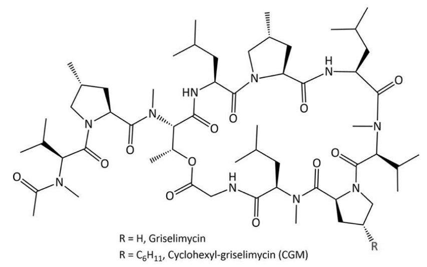

EC/11716

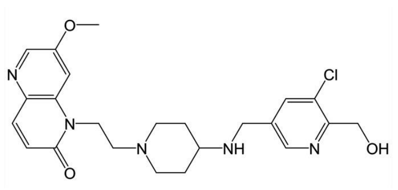

Protein synthesis

EC/11770

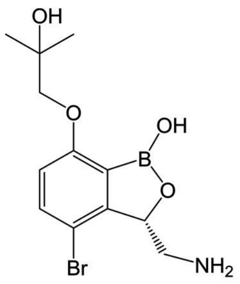

Epetraborole

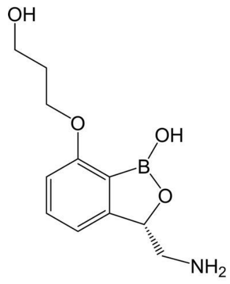

LCB01-0371

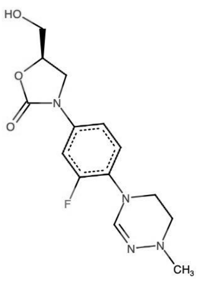

Energy metabolism

TBAJ-876

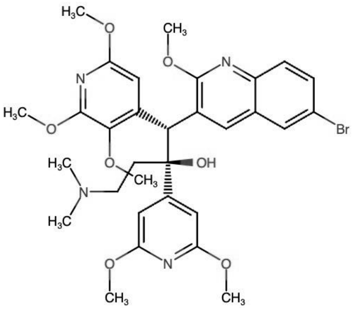

WX-081

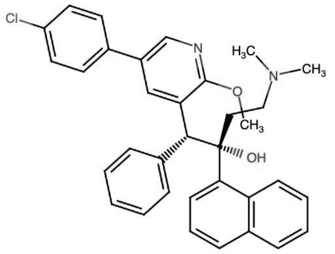

Other mechanisms

MD3

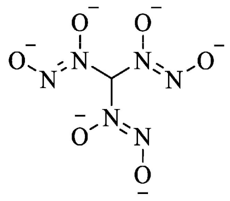

AR-12

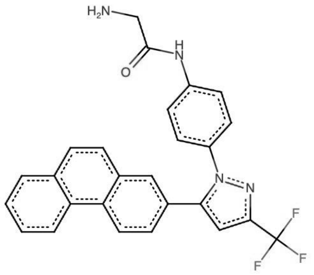

OZ439

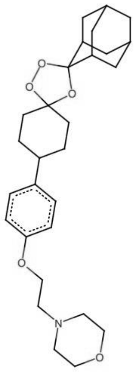

Supplement: Supplementary file 1 [file Data_Sheet_1.pdf]
